# Oestrogenic Activity in Girls with Signs of Precocious Puberty as Exposure Biomarker to Endocrine Disrupting Chemicals: A Pilot Study

**DOI:** 10.3390/ijerph20010014

**Published:** 2022-12-20

**Authors:** Marta Gea, Anna Toso, Giuseppe Nicolò Bentivegna, Raffaele Buganza, Enrica Abrigo, Luisa De Sanctis, Tiziana Schilirò

**Affiliations:** 1 Department of Public Health and Pediatrics, University of Turin, 10126 Turin, Italy; 2 Institut de Recherche en Cancérologie de Montpellier (IRCM), Inserm U1194, Institut régional du Cancer de Montpellier (ICM), Université Montpellier, 34090 Montpellier, France; 3 Unit of Pediatric Endocrinology, Department of Public Health and Pediatrics, Regina Margherita Children Hospital, University of Turin, 10126 Turin, Italy

**Keywords:** early puberty, endocrine disruptors, exposure biomarker, MELN gene reporter assay, oestradiol equivalent concentration, urban environment

## Abstract

The relationship between endocrine disrupting chemical (EDC) exposure and Precocious Puberty (PP) was investigated in this pilot study, involving girls with signs of PP (P) and pre-pubertal girls (C). Risk factors for PP were assessed through questionnaires, while 17β-oestradiol (E2) levels and oestrogenic activity were quantified on sera. The oestrogenic activity, expressed as E2 equivalent concentration (EEQ), was applied as EDC exposure biomarker. Questionnaires showed a low EDC knowledge, a high EDC exposure, and a potential relationship between some habits at risk for EDC exposure and PP. EEQs were similar between C and P; however, they were significantly higher in girls living in an urban environment than in girls living in a rural environment, suggesting a potential higher EDC exposure in cities. The results of this pilot study highlighted the need to raise awareness on EDCs and can be considered a starting point to clarify the relationship between EDC exposure and PP.

## 1. Introduction

Puberty represents an essential step in the dynamic transition from childhood to adulthood that leads to full reproductive capacity. The mechanisms underlying this process have not been fully understood and include genetic, metabolic, environmental, geographic, and nutritional factors. Indeed, it has been reported that puberty can be influenced by adipose tissue, gastrointestinal function, adrenal androgen production, physical, and psychological stress [1,2,3,4,5]. 

Precocious Puberty (PP) is an early onset of puberty and is defined as the development of secondary sexual characteristics before the age of eight years in girls and nine years in boys. This development is clinically assessed through Tanner Staging, a rating based on visual estimates using a scale from one (no development) to five (adult maturity) [6,7,8].

Although PP can be related to tumours involving the hypothalamic-pituitary-gonadal axis (HPG axis), genetic factors or rare syndromes, most PP cases are idiopathic, so the risk factors influencing the onset of PP in children have not been fully clarified. Environmental factors may play a role in the timing of pubertal development such as general health status, nutrition, environmental conditions, and socioeconomic status [9,10]. Furthermore, recent studies reported a correlation between the amount of body fat and PP [5,11,12,13]. Not only being overweight and/or obese may influence the onset of PP but also diet, since a high-fat and/or high-glycaemic-index diet may induce a low-grade hypothalamic inflammation and subsequent premature activation of the gonadotropin releasing hormone [5]. A shorter duration of breastfeeding has also been correlated with PP and a relationship between breast milk assumption during the neonatal period and prevention of both overweight and PP has been reported [14]. Recently, the review of Spaziani et al. [8] suggested that early or delayed pubertal development could be related to a group of heterogeneous molecules called endocrine disrupting chemicals (EDCs).

EDCs are exogenous substances or mixtures that alter the function of the endocrine system and consequently cause adverse health effects in an organism or its progeny [15]. They interact with the organism through multiple mechanisms such as direct interaction with the hormonal receptors, interference with the post-receptor pathways, and suppression of hormonal synthesis. Regarding their influence on puberty, their androgenic and oestrogenic effects are crucial, as they could cause an interference with the HPG axis [8]. EDCs can have a natural origin, such as sex steroids, phytoestrogens, and mycotoxins, or a synthetic origin, such as the compounds used in manufacturing (e.g., phthalates, phenols, polychlorinated biphenyls, and polycyclic aromatic hydrocarbons), in the pharmaceutical industry (e.g., 17α- ethinyloestradiol and diethylstilbestrol), or in agriculture (pesticides such as DDT, atrazine, and glyphosate). They are widely spread in the environment, since their presence has been reported in aquatic environments [16,17,18,19], in tap water [20], and in indoor and outdoor air [21,22,23,24]. Finally, at low concentrations, they are present in a multitude of everyday products, such as personal care products and cosmetics [25]. Sources of EDC exposure are numerous and very diverse (e.g., air, water, soil, food, everyday products) and humans are exposed to EDCs not only outdoors, but also in living and working environments. Once entered in the body through ingestion, inhalation, or skin contact, EDCs can reach the bloodstream and can be spread throughout the body [15,26]. Therefore, human EDC exposure greatly varies depending on habits, age, living and working environments. 

Due to their behaviour and their peculiar metabolic characteristics, children are particularly exposed to EDCs [15,22] and can have higher concentrations of EDCs in their biological matrices than adults. For example, Rose et al. [27] found that polybrominated diphenyl ether levels were 2 to 10 times higher in children than in adults.

Due to multiple chemicals acting as EDCs, the different routes of exposure and the different factors influencing exposure, the assessment of overall exposure to EDCs is very complex. Using traditional monitoring methods (i.e., chemical analysis), it is only possible to assess a few EDCs in biological matrices and the combined effect of different EDCs cannot be estimated. Therefore, novel methods to assess EDC exposure were proposed by the scientific community. These methods detect the biological effect induced by a mixture of EDCs rather than quantifying the presence of individual EDCs. In particular, oestrogenic activity assays have been proposed to assess EDC exposure in biological samples [28]. 

The aims of this pilot study were (i) to investigate the possible relationship between signs of PP and potential risk factors (e.g., exposure to EDCs in daily life, occupational EDC exposure of parents, eating habits), (ii) to evaluate the potential application of an in vitro assay (i.e., the gene reporter luciferase assay) as a biomarker of exposure to EDCs. The study involved 40 subjects: girls with clinical signs of PP were classified as cases (P), while age-matched pre-pubertal girls were classified as controls (C). The association between signs of PP and potential risk factors for its occurrence was investigated through questionnaires completed by girls’ parents. Moreover, girls’ serum samples were collected and were analysed with both a chemiluminescence immunoassay to quantify 17β-oestradiol (E2) and a gene reporter luciferase assay to assess the oestrogenic activity. This study was carried out during an exceptional period characterised by the COVID-19 pandemic, that negatively affected the study course (e.g., complications in the recruitment and interruption of research activities) reducing the sample size. 

## 2. Materials and Methods

### 2.1. Study Design

This pilot study was approved by the Ethical Committee of the A.O.U. Città della Salute e della Scienza di Torino—A.O. Ordine Mauriziano—A.S.L. Città di Torino and involved 40 girls aged between 3 and 8 years. The subjects were recruited from the Regina Margherita Children Hospital of Turin (Piedmont region, Northern Italy). The parents of girls provided written informed consent to participate in the study. 

Girls with clinical signs of PP confirmed during their access to the paediatric endocrinology clinic were included in P (*n* = 30). According to the clinical–hormonal examinations, 13 girls were affected by central PP (characterized by activation of the HPG axis), while 17 were affected by incomplete PP (without activation of the HPG axis). At the time of diagnosis, the stages of breast development according to the female breast development scale of Tanner were the following. Among girls with central PP, 5 girls were classified as stage II, while 8 girls as stage III; among girls with incomplete PP, 12 girls were classified as stage II, while 5 girls as stage III. Regarding Tanner stages of pubic hair development, among girls with central PP, 10 girls were classified as stage I, 2 girls as stage II, and a girl as stage III; among girls with incomplete PP, 10 girls were classified as stage I, 5 girls as stage II, and 2 girls as stage III. One girl with central PP and one with incomplete PP had one episode of blood spotting reported at diagnosis, but not clear menarche. Genetic analyses for PP predisposition were not carried out for this study. At the time of diagnosis and of blood sampling, no P girls had medical treatment for PP, which was subsequently started in 12 out of 13 patients with central PP.

Age-matched pre-pubertal girls were included in C (*n* = 10). Exclusion criteria were: age ≥8 years, male sex, and presence of endocrine disorders (for C). 

In order to collect information on the study population and to evaluate the association between signs of PP and potential risk factors for this condition, questionnaires were distributed to girls’ parents. Moreover, blood samples were collected, and sera were analysed through a chemiluminescence immunoassay to quantify E2 and through a gene reporter luciferase assay to assess the oestrogenic activity as a biomarker of exposure to EDCs. 

### 2.2. Questionnaires

Questionnaires were composed of both open and closed-ended questions and they were organized in the following sections: children personal data (e.g., age, weight, height, residence, clinical data), information about mother and father (e.g., educational level, EDC knowledge), pregnancy (e.g., personal and clinical data during pregnancy), neonatal age (e.g., childbirth type, clinical data during neonatal age, breastfeeding type), type of pubertal development of parents and of any other siblings, possible EDC exposure due to residence, parents’ occupation, lifestyle, and eating habits (see Supplementary Materials 1). Data collected through questionnaires were entered into a database using Microsoft Excel, anonymising the participants to preserve privacy during the study.

#### Data Processing

Weight and height data were used to calculate the children’s body mass index (BMI) (weight [kg]/height [m] squared) which was used to classify the children as underweight, normal weight, overweight, or obese according to the BMI cut-offs for age proposed by Cole et al. [29,30].

To assess the level of total exposure to EDCs, the sum of residence-related exposure factors (maximum 5 factors) and lifestyle-related exposure factors (maximum 13 factors) was calculated. These two types of factors were chosen as representative of a “current” exposure, while other exposure factors were not considered (e.g., exposure during pregnancy or neonatal period). Therefore, the minimum level of exposure was equal to 0 (no exposure factor), while the maximum level of exposure was equal to 18 (all exposure factors due to residence and lifestyle). The EDC cumulative exposure level of each girl was defined as “low” when exposure factors ranged between 1 and 6 or “high” when exposure factors ranged between 7 and 18. The threshold of 6 was chosen based on the results obtained from the questionnaires: 6 corresponds to half of the maximum number of exposure factors found in this study for a single girl (maximum exposure factors = 11). 

### 2.3. Chemiluminescence Immunoassay (E2 Concentrations)

A chemiluminescence immunoassay was applied in order to quantify E2 concentrations in serum samples using the Elecsys Estradiol III kit (Roche Italia S.p.A., Monza, Italy). The assay was carried out according to manufacturer instructions. The detection limit of the assay was equal to 5 pg/mL.

### 2.4. Gene Reporter Luciferase Assay (Oestrogenic Activity)

The oestrogenic activity of serum samples was assessed using a gene reporter luciferase assay which measures the expression of a reporter gene regulated by oestrogens [31]. The assay was carried out using the luciferase-transfected MELN cells kindly provided by Dr. P. Balaguer (INSERM, Montpellier, France), which derive from MCF7 breast cancer cells after transfection with the ERE-βGlob-Luc-SVNeo plasmid. The method was applied using the protocol reported by Schilirò et al. [32] modified according to a literature review [28]. MELN cells were grown in 75 cm^2^ flasks in a 5% CO_2_/95% air atmosphere under saturating humidity at 37 °C. For cell growth Dulbecco’s Modified Eagle’s Medium Nutrient Mixture F12 Ham (DMEM-F12) medium was used; the medium was supplemented with phenol red and the following supplements: 5% *v*/*v* of Foetal Bovine Serum (FBS), 4 mM of L-Glutamine, 100 U/mL–100 μg/mL of Penicillin/Streptomycin, and 1 mg/mL of G418 Geneticin. Since this culture medium can cause oestrogenic activity and impair the results, DMEM-F12 without phenol red and with stripped FBS (i.e., FBS treated with charcoal and dextran in order to remove oestrogens) was used as the experimental medium. The stripped FBS was purchased by Merck (Darmstadt, Germany). Three days before the experiment, the culture medium of 75 cm^2^ flasks containing cells was replaced with experimental medium to allow cells to adapt. For the experiment, cells of the adapted flasks were trypsnised, counted with hemocytometer (Bürker counting chamber), and seeded in white 96 wells-plates at a density of 40,000 cells/well in 100 μL/well of experimental medium. Cells were then incubated in a 5% CO_2_/95% air atmosphere under saturating humidity at 37 °C. After 24 h, the experimental medium was removed and replaced with 100 μL of each sample dilution for three replicas. Each human serum sample was diluted at 5% *v*/*v* in experimental medium, adding 5% *v*/*v* of stripped FBS in order to reach a final concentration of 10% of serum (human serum + stripped FBS). Thereafter, cells were incubated again for 21 h. In each plate, a negative control (C−), which consists of experimental medium supplemented with stripped FBS (10% *v*/*v*), and a positive control (C+), which corresponds to E2 in a concentration of 10^−8^ M in experimental medium supplemented with stripped FBS (10% *v*/*v*) were included. Moreover, for each independent experiment, a standard response curve of E2 was set up. The response curve was made up of increasing E2 concentrations in experimental medium with 10% of stripped FBS (final E2 concentrations from 10^−12^ M to 10^−8^ M). Each treatment was tested in triplicate in two independent experiments. After 21 h incubation, 100 μL of One-Glo Luciferase Assay System (Promega Italia S.r.l., Milan, Italy), containing fluoroluciferin were added in each well. Plates were shaken for 5 min to allow complete cell lysis. Within 30 min after cell lysis, the luminescence of each well was measured with a luminometer (Infinite Reader M200 Pro, Tecan, Männedorf, Switzerland). 

The oestrogenic activity was expressed as relative luciferase activity and was calculated as a percentage of the activity induced by the treatment with respect to the activity induced by the positive control, subtracting the activity of the negative control (relative luciferase activity of C− = 0%, relative luciferase activity of C+ = 100%). The results were elaborated and expressed as E2 equivalent concentration (EEQ). The EEQ is defined as the total concentration of all oestrogenic active compounds in sample normalised to the E2. To express the data as EEQ using statistical analysis software (IBM SPSS Statistics 25, IBM, Armonk, NY, USA), the dose–response curve equation of the reference compound (E2) was estimated. The equation was estimated by entering the logarithm in base ten of the molar concentrations of E2 tested as the independent variable and the relative luciferase activity induced by each concentration as the dependent variable (best fitting = quadratic function; R^2^ = 0.94, *p* < 0.001). Next, the relative luciferase activity of the samples tested at 5% was interpolated onto the function and the EEQ value of each sample was thus obtained [33]. Since each sample was tested in two independent experiments, the final EEQ of each sample was expressed as the mean ± standard deviation of the EEQs obtained in the two experiments. The sensitivity of the reporter gene assay on MELN cells is 0.3 pg/mL. However, since the serum samples were diluted to 5% in the experimental culture medium, the sensitivity under the experimental conditions was 6.5 pg/mL.

### 2.5. Data Analysis and Statistical Analysis

Statistical analyses of data obtained from questionnaires and from laboratory analyses (E2 concentrations and oestrogenic activity) were performed using IBM SPSS Statistics (IBM, Armonk, NY, USA). To summarize general information of the study population, data collected from questionnaires were analysed with descriptive statistics and compared between C and P to investigate a possible association between signs of PP and risks factors (e.g., EDC exposure). Regarding laboratory analyses, as for questionnaires, mean E2 concentrations and mean EEQs were calculated for the whole population and then compared between C and P. Finally, mean E2 concentrations and mean EEQs were compared considering the level of EDC exposure (low or high) and the living environment (urban or rural). For data analysis, E2 concentrations below the detection limit (<5 pg/mL) were considered equal to half the detection limit of the assay (2.5 pg/mL). The Shapiro–Wilk test was performed in order to evaluate data distribution of continuous variables. Since data were not normally distributed, the mean values of these variables were compared among the groups using non-parametric tests (i.e., Mann–Whitney test). Finally, Chi-Square test was performed in order to compare the distribution of categorical variables among the groups. The results were considered statistically significant for *p* ≤ 0.05. 

## 3. Results and Discussion

### 3.1. Questionnaire Results

#### 3.1.1. General Characteristics

The study population was composed of 40 female children (three–eight years of age). According to residence, 30.0% of the study population (*n* = 12) lived in Turin (the main city of Piedmont region), 45.0% (*n* = 18) in Turin province, and 25.0% (*n* = 10) in other provinces of the Piedmont region (Northern Italy). The average age was 7.34 ± 1.17 years, while the average BMI was 16.40 ± 3.06 kg/m^2^.

Based on the specific BMI cut-offs for age proposed by Cole et al. [29,30], 73.0% of girls (*n* = 27) were normal weight, 13.5% (*n* = 5) were underweight, and 13.5% (*n* = 5) were overweight or obese. For three girls it was not possible to calculate BMI due to missing data in the questionnaires. The percentage of overweight/obese girls was lower than the national data reported by the study of Lauria et al. [34]. This study reported the national data on the prevalence of overweight and obese children collected in the national study “OKkio alla SALUTE”, part of the WHO/Europe Childhood Obesity Surveillance Initiative (COSI); it stated that, during year 2016, the prevalence of overweight children (including obese children) among primary school girls was at 30.5% considering the population residing in the whole of Italy, while it was 25.7% considering the population residing in Northern Italy [34]. 

Regarding parents’ educational level, 28.2% of the mothers and 29.7% of the fathers had an eighth grade or professional diploma, 41.0% and 56.8% had a high school diploma, and 30.8% and 13.5% had a degree.

Concerning EDC knowledge, only 28.2% of the parents declared to know them, while the remaining proportion stated that they had never heard of them (71.8%). EDC knowledge was mainly due to information acquired by TV, internet, books and articles, medical doctors, or work colleagues. This result suggested that it would be extremely important to raise awareness about EDCs and their possible impact on human health. Information campaigns would be especially useful among parents-to-be, since prenatal and early childhood exposure to EDCs may greatly affect the proper development of the child [35,36]. 

#### 3.1.2. General Characteristics: Comparison between C and P 

Mean age was 6.30 ± 1.34 years for C (min 3.90 years, max 7.70 years) and 7.49 ± 0.70 years for P (min 4.90 years, max 7.90 years). A statistically significant difference was found between the two groups (Mann–Whitney test, *p* < 0.001).

Mean BMI was 15.83 ± 4.99 kg/m^2^ for C (min 10.42 kg/m^2^, max 27.78 kg/m^2^) and 16.58 ± 2.22 kg/m^2^ for P (min 13.66 kg/m^2^, max 24.49 kg/m^2^). Although mean BMI was not statistically different between C and P (Mann–Whitney test, *p* > 0.05), the percentages of normal weight, underweight, and overweight/obese girls were statistically different (Chi-Square test, *p* = 0.003); in particular, the percentage of overweight/obese girls was higher in C than in P. This result was unexpected as being overweight/obese has been positively associated with the onset of PP [12,14,37]. Possible interpretations of this result could be the awareness of parents that eating habits can affect the onset and development of this condition (e.g., information received from the general practitioner) and/or the little case series of the present study. 

Mothers’ and fathers’ pubertal development and familiarity for PP were not statistically different between C and P. On the contrary, a statistically significant difference was found between C and P in the percentage of siblings with signs of PP. Indeed, the girls in C had no siblings with signs of PP, whereas 75.0% of the girls in P had at least one sibling affected by the same condition (Chi-Square test, *p* = 0.005). This result is interesting as it confirmed a genetic predisposition to PP, already reported in literature [38,39,40], but could also suggest the importance of lifestyle habits adopted by families (i.e., due to lifestyle habits, all the children of a single family could be highly exposed to EDCs, thus may be more at risk for PP). 

Considering the living environment (urban or rural), the percentage of girls living in an urban environment was higher, although not statistically significant, in P than in C (40.0% and 20.0%, respectively).

#### 3.1.3. EDC Exposure 

The results showed that a high percentage of parents (especially fathers) were potentially exposed to EDCs in their workplace. Indeed, 42.1% of fathers and 15.4% of mothers worked in the agriculture sector, rubber industries, plastic industries, petrochemical plants, metallurgical plants, electronic industries, beauty sectors, dye industries, and incinerators, which can be considered sources of exposure to EDCs [15]. Moreover, 67.5% of families lived near one or more potential sources of EDCs (i.e., near an industrial plant, mechanical workshop, landfills, cultivated field, chemical warehouse) [15].

Possible exposure to EDCs due to lifestyle was also assessed considering 13 habits that can cause exposure to EDCs. For example, the use of plastic bottles, plastic containers, and clingfilm were considered habits at risk. Indeed, EDCs are contained in these products (e.g., phenols and phthalates) and can be transferred especially to warm foods [41,42]. Consumption of packaged meat/vegetables was also considered a habit at risk as it can be a source of exposure to phthalates and bisphenols [43,44]. In addition, the use of personal care products, which may contain phthalates and parabens [45], was evaluated as a habit that can increase EDC exposure [15]. 

Overall, girls seem to be potentially exposed to EDCs as 100% of families adopt at least one lifestyle habit that could increase EDC exposure. In particular, the most adopted habits were the use of care creams, the use of clingfilm, the use of spray cosmetics, and the consumption of packaged meat/vegetables. 

Through questionnaires, the possible exposure to EDCs through eating habits was also evaluated. Several studies seem to suggest a correlation between eating habits, such as the consumption of canned or highly processed food, and the intake of EDCs. These products may contain several EDCs (e.g., bisphenol A) that can be transferred into food [46,47,48,49]. Consumption of water in plastic bottles has also been associated with exposure to EDCs (e.g., phenols, phthalate) [20,41,42,50]. Moreover, some cooking techniques, due to high temperatures, could lead to food carbonization with the consequent formation of polycyclic aromatic hydrocarbons [15,47,48,51,52]. Finally, consumption of soy products in childhood has also been considered to cause a possible increase in EDC exposure (i.e., phytoestrogens) and was associated with PP in girls [53]. 

The study population also appears to be potentially exposed due to eating habits. Indeed, a high percentage of families declared a weekly consumption of water in plastic bottles (87.2%), of grilled food (42.5%), and of canned food (34.2%). 

#### 3.1.4. EDC Exposure: Comparison between C and P 

Regarding EDC exposure due to lifestyle, 4 out of 13 habits were found to be significantly different between C and P. As reported in Table 1, the use of disposable plastic containers, the storage of warm foods in plastic containers, the consumption of packaged meat/vegetables, and the use of candles/incense/air fresheners were significantly higher in C than in P. Considering that the adoption of these habits may lead to a greater exposure to EDCs [43], overall, the results suggested that girls in P were less exposed to EDCs than girls in C. This unexpected result could be related to the awareness of the parents that eating habits can affect the onset and development of this condition and/or the little case series of the current study, so it should be further investigated. 

Since exposure by ingestion represents one of the main routes of EDC exposure for humans, the potential association between eating habits and the development of signs of PP was investigated comparing C and P. A total of 8 out of 18 eating habits were found to be significantly different between C and P (Table 2). 

In particular, a higher consumption (in terms of frequency) of water in glass bottles and of water from public sources was found in C with respect to P. These results are interesting, because they can suggest a potential relationship between EDC exposure and signs of PP. Indeed, a greater consumption of water in glass bottles leads to a lower consumption of water in plastic which can instead lead to exposure to EDCs such as, for example, phenols and phthalates. Regarding the consumption of water from public sources, although it does not directly lead to a lower exposure to EDCs, this behaviour could be associated with the use of non-disposable glass bottles. In fact, the consumption of water from public sources often takes place using glass bottles while generally those who consume water already bottled buy it in disposable plastic bottles (more easily available on the market and at a lower cost).

The consumption of pork, sheep, and other meat types was more frequent in C than in P. This result is unexpected since it has been reported that the intake frequency of bovine, sheep, and pork meat were inversely associated with the age at menarche [54] and the consumption of poultry, pork, and processed meat was positively associated with the levels of some EDCs in human serum [27]. However, it is important to underline that the relationship between meat intake, EDC exposure, and age at menarche can be affected by numerous factors. For example, it is not easily identifiable which meat type can lead to a higher EDC exposure, representing a potential risk factor for PP. In fact, the presence of EDCs in meat can depend on many factors including the characteristics of the animal (e.g., species, sex, age that can influence the production of endogenous steroid hormones), materials used to process and package meat, the possible use of hormones in the breeding phases (although Europe banned meat from animals treated with steroid growth hormones), and the use of cooking techniques that can favour EDC presence in foods (e.g., grilling). Moreover, beside EDC-related mechanisms, meat intake might induce an earlier age at menarche through, for example, development of adiposity or micronutrient availability [54].

Conversely, questionnaire results showed that the frequency of canned food consumption was higher in P than in C. This result could suggest that EDC exposure through this eating habit could be associated with the occurrence of PP.

Finally, regarding food cooking techniques, a higher frequency of grilling and frying was found in C than in P. This result is unexpected as overcooking food on the grill could lead to EDC exposure [47,51,52] and is also opposite to the study of Chen et al. [55], in which an unhealthy diet pattern, heavy in fried food, was significantly positively associated with PP.

Overall, the percentage of girls with a high EDC exposure was higher, although not significant, in C than in P (70.0% and 40.0%, respectively).

### 3.2. Results of Laboratory Analyses

#### 3.2.1. E2 Concentrations

**Figure 1:**
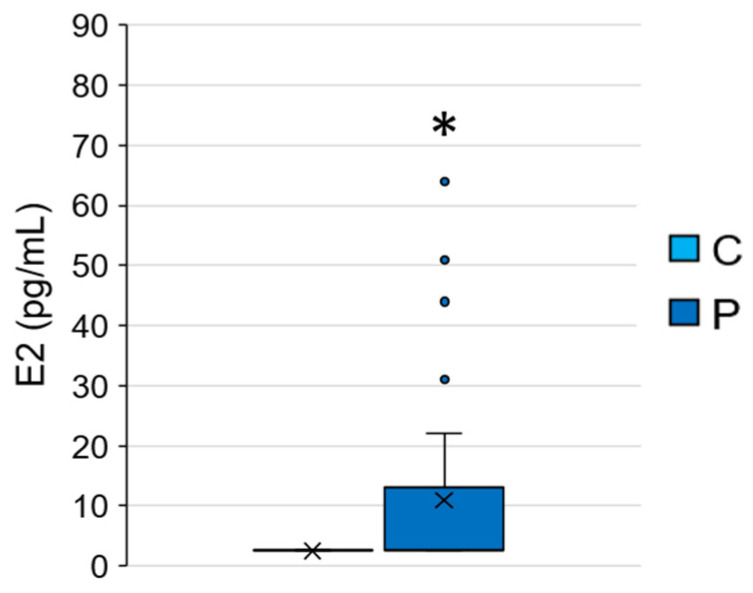
17β-oestradiol (E2) concentrations measured in C and P. * = *p* ≤ 0.05, Mann–Whitney test.

The average E2 concentration of the 40 subjects was 8.85 ± 14.43 pg/mL (min < detection limit, max 64.00 pg/mL), and the comparison between C and P showed that E2 concentrations were significantly higher in P than in C (P = 10.97 ± 16.17 pg/mL, C ≤ detection limit, Mann–Whitney test, *p* = 0.040) (Figure 1). Moreover, regarding P, the average E2 concentration of girls with central PP was 18.50 ± 21.97 pg/mL, while that of girls with incomplete PP was 5.21 ± 5.50 pg/mL. This result was expected since signs of PP are usually the clinical feature of a premature activation of the HPG axis, which leads to E2 release in females resulting in a high concentration of this hormone in their blood [1,56,57,58], but could also be due to other external factors such as EDC exposure [8,59].

The comparison between E2 concentrations found in the present study with the physiological E2 concentrations during the pre-pubertal period showed that the average E2 concentrations in the serum of girls in the present study were within the range of the physiological values (<27 pg/mL, data provided by the Regina Margherita Children hospital, Table S1 in Supplementary Materials 2). As expected, some E2 concentrations measured in P were higher than the upper limit of the pre-pubertal physiological range, confirming that hormone concentrations can be high in these girls. 

#### 3.2.2. EEQ Concentrations

The average EEQ concentration of the 40 subjects was 16.62 ± 15.58 pg/mL (min 6.10 pg/mL, max 80.10 pg/mL). Table 3 shows the mean EEQ concentrations of C and P in comparison with the mean EEQ concentrations of pre-pubertal girls and of PP girls reported in previous studies [60,61,62,63,64,65,66,67,68]. As can be observed, the mean EEQ concentration of C found in the present study (21.03 ± 21.41 pg/mL) was higher than EEQ concentrations found in literature. This finding could suggest that girls in C may have a higher EDC exposure than pre-pubertal girls analysed by other studies. Moreover, EEQ concentration of P found in the present study (15.14 ± 13.24 pg/mL) was similar to or higher than the EEQ concentrations reported by previous studies [61,63,65,69]. It is important to underline that the discrepancy among results could be due to the different methodologies applied in order to estimate EEQ by the different authors [28]. 

**Figure 2:**
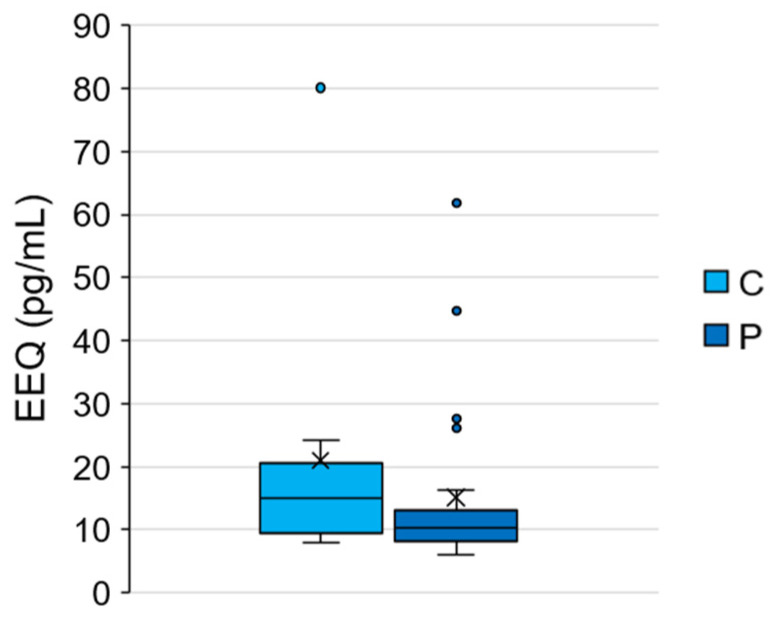
EEQ concentrations measured in C and P. Data were not statistically different (Mann–Whitney test, *p* > 0.05).

Unlike E2 concentrations, no significant differences were found between C and P in EEQ concentrations (Mann–Whitney test, *p* > 0.05) (Figure 2). Therefore, the present study did not find a strong relationship between EDC exposure and signs of PP. However, some confounding factors could have influenced the results. For example, our control group seems to be highly exposed to EDCs compared to the general population. Indeed, a higher EEQ was found in C with respect to the literature and a higher EDC exposure in C than in P was also confirmed through the questionnaire analysis (e.g., the percentage of highly exposed girls was higher, although not significant, in C than in P: 70.0% and 40.0%, respectively).

#### 3.2.3. Relationship between EDC Exposure (Low/High) and EEQ

To evaluate the relationship between EDC exposure and oestrogenic activity, the EEQ was compared according to the level of cumulative exposure to EDCs derived from questionnaires. In particular, the oestrogenic activity was compared between girls with a low EDC exposure (*n* = 21) and girls with a high EDC exposure (*n* = 19). In order to consider the activity due to endogenous hormones, E2 concentrations were also compared between the two groups.

**Figure 3:**
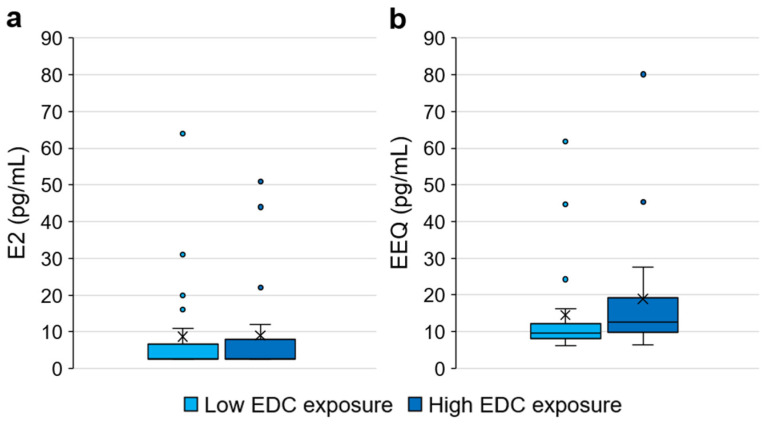
Comparison between girls with low and high endocrine disrupting chemical (EDC) exposure: (**a**) E2 concentrations; (**b**) EEQ concentrations. Data were not statistically different (Mann–Whitney test, *p* > 0.05).

No statistically significant difference was detected between the two groups (i.e., low/high EDC exposure) for both the considered parameters (i.e., E2 and EEQ) (Mann–Whitney test, *p* > 0.05) (Figure 3). However, it is interesting to note that while the E2 concentrations seem similar in the two groups (mean E2: low EDC exposure = 8.67 ± 14.76 pg/mL, high EDC exposure = 9.05 ± 14.45 pg/mL), in the low EDC exposure group, a lower EEQ was detected than in the high EDC exposure group (mean EEQ: low EDC exposure = 14.55 ± 13.72 pg/mL, high EDC exposure = 18.89 ± 17.51 pg/mL). This result, despite not significant, could suggest that, with the same endogenous hormone levels (i.e., E2 concentrations) in the high EDC exposure group, a higher concentration of oestrogenic compounds may be present in the serum, resulting in a higher oestrogenic activity. Therefore, this study showed a partial concordance between EDC exposure (estimated using questionnaires) and the results of the gene reporter luciferase assay (applied as a biomarker of EDC exposure). The discrepancy between EDC exposure assessed by questionnaires and EEQ could be explained considering that EDCs are heterogeneous and ubiquitous chemicals whose assessment is difficult to estimate using only questionnaires. Moreover, it should be considered that, through EEQ, only EDCs with oestrogenic activity can be quantified.

#### 3.2.4. Relationship between Living Environment (Rural/Urban) and EEQ

Oestrogenic activity was also compared according to the type of living environment (urban or rural environment). In particular, oestrogenic activity was compared between girls residing in a rural environment (*n* = 26) and girls residing in an urban environment (*n* = 14). In order to consider the activity due to endogenous hormones, E2 concentrations were also compared between the two groups.

**Figure 4:**
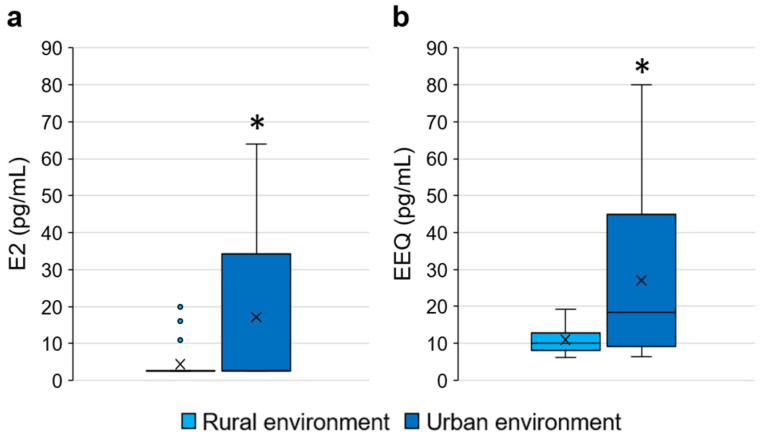
Comparison between girls living in a rural environment and girls living in an urban environment: (**a**) E2 concentrations; (**b**) EEQ concentrations. * = *p* ≤ 0.05 Mann–Whitney test.

Th average E2 concentration in the rural environment was 4.38 ± 4.73 pg/mL (min 2.50 pg/mL, max 20.00 pg/mL), while in the urban environment it was 17.14 ± 21.62 pg/mL (min 2.50 pg/mL, max 64.00 pg/mL); average EEQ concentration in the rural environment was 10.98 ± 3.59 pg/mL (min 6.10 pg/mL, max 19.20 pg/mL), while in the urban environment it was 27.08 ± 22.86 pg/mL (min 6.40 pg/mL, max 80.10 pg/mL). Statistically significant differences were found between two groups (i.e., rural vs. urban environment) for both the considered parameters (Mann–Whitney test- E2: *p* = 0.031; EEQ: *p* = 0.020) (Figure 4).

This result suggests that living in urban or industrial areas could cause higher oestrogenic activity that is probably due to a higher exposure to EDCs. This hypothesis is supported by several studies which show that people living in urban environments can be exposed to EDCs. The exposure may occur through polluted and heavily anthropized rivers and through the consumption of tap water. Indeed, some studies have shown that the presence of EDCs in anthropized watercourses, due to inadequate urban wastewater management, can lead to pollution of drinking water sources. Since drinking water treatment technology can be ineffective in removing these compounds, consumption of tap water can cause the consequent EDC intake [18,20,70]. Exposure in urban areas may also occur through inhalation of atmospheric EDCs released by human activities (e.g., traffic, industrial processes, waste incineration) [21,71].

In addition to higher oestrogenic activity in girls living in an urban environment than in a rural one, the results of the present study showed that the percentage of girls living in an urban environment was higher, although not statistically significant, in P than in C (40.0% and 20.0%, respectively). This finding suggested that urban environments might influence the age at menarche. In accordance with this hypothesis, several studies reported a significant association between residence in areas with high traffic or high air pollution, such as urban areas, and an earlier age at menarche [72,73,74]. 

However, although people living in urban environments can be highly exposed to EDCs, an exposure can occur also in rural environments due to agriculture (i.e., use of pesticides in fields) [15] or lifestyle habits; indeed, according to questionnaire results, the percentage of girls living in a rural environment that have a high exposure to EDCs was 57.69%, while the percentage of girls living in a urban environment that have a high exposure to EDCs was only 28.57%.

### 3.3. Study Limitations

The present study was affected by some limitations. In particular, the applied method (i.e., gene reporter luciferase assay) assessed the overall oestrogenic activity but was not suitable to distinguish between the activity induced by EDCs from that of endogenous hormones. Although this disadvantage was resolved by evaluating the concentration of the main endogenous hormone in the serum (i.e., E2), the interpretation of the results is quite complex. Moreover, despite the estimate of EDC exposure through questionnaires was crucial to integrate the laboratory analysis results, it is important to underline that it was not simple to assess EDC exposure levels through this method. Indeed, EDCs are a group of very heterogeneous molecules that are widely present in numerous everyday products and environmental matrices, so the sources of exposure can be many and estimating the realistic levels of exposure to EDCs is very complex. Therefore, to better estimate EDC exposure, in future studies, it could also be interesting to perform a chemical analysis on serum samples in order to quantify some specific EDCs (e.g., bisphenol A, phthalates, parabens). 

This study was carried out during an exceptional period characterised by the COVID-19 pandemic. For more than a year, in hospitals, priority was given to more severe health conditions; therefore, paediatric endocrinology accesses due to suspected PP signs were reduced. The pandemic and the subsequent slow return to normality also caused a lower rate of adhesion in the study by the girls’ families. Therefore, this emergency period has affected the project concerning subject recruitment; furthermore, it has impeded the study course as research and laboratory activities at the university were interrupted due to lockdowns. Consequently, the results of this study may be influenced by sample size, so in order to confirm the results, it would be important to perform other studies on larger populations. 

Despite limitations, this study evaluated the possible association between an important clinical condition (i.e., signs of PP), whose incidence is increasing [4,75,76,77], and the exposure to environmental contaminants of emerging concern (i.e., EDCs). The association was studied through the application of an effect-based monitoring tool (i.e., gene reporter luciferase assay) that has the advantage to potentially assess the cumulative exposure to all the substances with an oestrogenic effect, also considering metabolites and substances whose oestrogenic activity has not been studied yet. 

## 4. Conclusions

This pilot study examined the relationship between signs of PP and potential risk factors including EDC exposure. Overall, the questionnaire analysis showed a low EDC knowledge and a high exposure through residence, lifestyle, and eating habits. The results also highlighted quite a high occupational exposure of parents. Since the lack of awareness about EDCs could lead to adoption of habits at risk, it would be important to raise awareness especially among future parents to avoid or decrease prenatal and early childhood exposure allowing the proper development of infants. 

As expected, laboratory results confirmed the premature activation of the HPG axis and the consequent E2 release in P (i.e., higher E2 concentration in P than in C).

Despite the comparison between C and P suggesting a potential relationship between some habits at risk for EDC exposure and signs of PP (i.e., to eat canned food and to drink less water in glass bottles and from public sources), a relationship between EDC exposure and signs of PP was not found. Therefore, it was not possible to confirm the interference of EDCs on HPG axis, nor their contribution to the occurrence of PP. This relationship might not be underlined due to potential confounding factors (e.g., the control group seems to be highly exposed to EDCs).

In addition, this study showed only a partial concordance between EDC exposure and the results of the gene reporter luciferase assay. The discrepancy could be explained considering that EDCs are heterogeneous and ubiquitous chemicals whose assessment is difficult to estimate using questionnaires and that EEQ only quantifies EDCs with an oestrogenic activity.

Finally, EEQ concentrations were significantly higher in girls living in an urban environment suggesting that these girls are potentially more exposed to EDCs than girls living in a rural environment. 

In conclusion, the results of this study suggested that the joint use of exposure information, quantification techniques, and biological effects-based tools might allow an integrated knowledge of different variables, being useful for the evaluation of a possible relationship between exposure to EDCs and PP. Since pubertal development is a complex process that includes genetic, environmental, and cultural factors, this pilot study can be considered a starting point for broader research aimed at clarifying the still existing doubts about the causes of PP in girls. 

## Figures and Tables

**Table 1:** Lifestyle habits for which a statistically significant difference was found between pre-pubertal girls (C) and girls with signs of PP (P). Data are expressed as percentages of families that adopt each habit.

	C (%)	P (%)	Chi-Square Test (*p*)
Use of disposable plastic containers	40.0	6.7	0.011
Storage of warm foods in plastic containers	90.0	33.3	0.002
Consumption of packaged meat/vegetables	80.0	43.3	0.044
Use of candles/incense/air fresheners	80.0	16.7	<0.001

**Table 2:** Eating habits for which a statistically significant difference was found between C and P. Data are expressed as percentages and are divided according to consumption frequency.

	Frequency	C (%)	P (%)	Chi-Square Test (*p*)
Consumption of sheep meat	Never	0.0	23.3	0.047
	Annually	0.0	20.0	
	Monthly	12.5	23.3	
	Weekly	87.5	33.4	
Consumption of pork meat	Never	0.0	13.3	0.022
	Annually	0.0	0.0	
	Monthly	10.0	46.7	
	Weekly	90.0	40.0	
Consumption of other meat *	Never	0.0	60.7	<0.001
	Annually	0.0	7.2	
	Monthly	0.0	21.4	
	Weekly	100.0	10.7	
Consumption of canned foods	Never	50.0	13.3	0.017
	Annually	12.5	0.0	
	Monthly	12.5	50.0	
	Weekly	25.0	36.7	
Consumption of water in glass bottles	Never	25.0	70.0	0.041
	Annually	0.0	0.0	
	Monthly	0.0	3.3	
	Weekly	75.0	26.7	
Consumption of water from public water sources	Never	14.3	82.8	0.002
	Annually	14.3	3.4	
	Monthly	0.0	0.0	
	Weekly	71.4	13.8	
Consumption of fried food	Never	0.0	3.3	0.011
	Annually	0.0	23.3	
	Monthly	10.0	43.4	
	Weekly	90.0	30.0	
Consumption of grilled food	Never	0.0	3.3	0.006
	Annually	0.0	23.3	
	Monthly	10.0	46.7	
	Weekly	90.0	26.7	

* meat other than bovine, sheep, pork, poultry.

**Table 3:** Serum 17β-oestradiol equivalent concentrations (EEQs) found in the present study in comparison with EEQ concentrations reported by previous studies for pre-pubertal girls and for PP girls. Data are presented as mean ± standard deviations (SD).

	Reference	Study Population (*n*)	EEQ (Mean ± SD, pg/mL)
Pre-pubertal girls	Present study	10	21.03 ± 21.41
	Klein et al., 1994 [62]	21	0.6 ± 0.6
	Klein et al., 1999 [61]	15	0.9 ± 1.0
	Paris et al., 2002 [66]	18	3.5 ± 2.2
	Larmore et al., 2002 [63]	12 ^a^	0.3 ± 0.4
	Larmore et al., 2002 [63]	12 ^b^	0.6 ± 1.3
	Wilson et al., 2003 [68]	34	3.4 ± 2.9
	Janfaza et al., 2006 [60]	147	1.6 ± 2.6
	Pereira et al., 2015 [67]	91	3.6 ± 2.3
	Mesa Valencia et al., 2019 [64]	107	3.6 ± 2.3
PP girls	Present study	30	15.14 ± 13.24
	Klein et al., 1999 [61]	20	2.3 ± 1.2
	Larmore et al., 2002 [63]	12	2.2 ± 2.7
	Gaspari et al., 2011 [69]	1	13.5 ± 1.0
	Paris et al., 2013 [65]	15	8.4 ± 7.3

^a^ = normal weight; ^b^ = obese.

## Data Availability

The data that supports the findings of this study are not publicly available due to privacy and ethical reasons.

## Supplementary Materials

The following supporting information can be downloaded at: https://www.mdpi.com/article/10.3390/ijerph20010014/s1, Questionnaire (Supplementary Materials 1), Table S1: physiological 17β-oestradiol (E2) concentrations (Supplementary Materials 2).
