# Supplementary material for: Oestrogenic Activity in Girls with Signs of Precocious Puberty as Exposure Biomarker to Endocrine Disrupting Chemicals: A Pilot Study
Source: Int J Environ Res Public Health. 2022 Dec 20;20(1):14. doi: 10.3390/ijerph20010014 (PMC9819927; doi:10.3390/ijerph20010014)
Supplement: Supplementary file 1 [file ijerph-20-00014-s001.zip › ijerph-2079944-supplementary.pdf]

SUPPLEMENTARY MATERIALS 1

Oestrogenic Activity in Girls with Signs of Precocious Puberty as Exposure Biomarker to Endocrine Disrupting Chemicals: A Pilot study

Marta **Gea**[1]*, Anna **Toso**[1,2], Giuseppe Nicolò **Bentivegna**[1], Raffaele **Buganza**[3], Enrica **Abrigo**[3], Luisa **De Sanctis**[1,3], Tiziana **Schilirò**[1]

[1]Department of Public Health and Pediatrics, University of Turin, 10126 Turin, Italy
[2]Institut de Recherche en Cancérologie de Montpellier (IRCM), Inserm U1194, Institut régional du Cancer de Montpellier (ICM), Université Montpellier, 34090 Montpellier, France
[3]Unit of Pediatric Endocrinology, Department of Public Health and Pediatrics, Regina Margherita Children Hospital, University of Turin, 10126 Turin, Italy

*Corresponding author:

Marta Gea

marta.gea@unito.it

Department of Public Health and Pediatrics,

University of Turin,

Via Santena 5 bis, 10126 Turin, Italy.

Phone: +39 0116703190

QUESTIONNAIRE

* = residence-related exposure factors that were used to assess the level of total exposure to EDCs.
+= lifestyle-related exposure factors that were used to assess the level of total exposure to EDCs.

SECTION 1 - Children personal data

Name:
Surname:
Residence:
Weight:
Height:
Age:

SECTION 2 - Information about mother and father

Mother educational level:
o 8th grade
o Professional diploma
o High school diploma
o Degree

Father educational level:
o 8th grade
o Professional diploma
o High school diploma
o Degree

Have you ever heard of endocrine disrupting chemicals?
o Yes
o No

SECTION 3 - Pregnancy

Did you have diseases during pregnancy?
o Yes
o No

Did you take drugs during pregnancy?
o Yes
o No

Did you have a pregnancy at term?
o Yes
o No

SECTION 4 - Neonatal age

Breastfeeding type:
o Breast milk
o False milk
o Mixed

Did you use plastic baby bottle during neonatal age?
o Yes
o No

Did your child have genital crisis during neonatal age?
o Yes
o No

Did your child have thelarche in the first 2 years of life?
o Yes
o No

SECTION 5 - Type of pubertal development of parents and any other siblings

Mother’s puberal development:
o Normal
o Precocious
o Tardive

Mother’s familiarity for precocious puberty:
o Yes
o No

Father’s puberal development:
o Normal
o Precocious
o Tardive

Father’s familiarity for precocious puberty:
o Yes
o No

Other children affected by precocious puberty?
o Yes
o No

SECTION 6 - Possible EDC exposure due to residence

Your home is located in close proximity to (less than 1 km):

Industrial plants*
o Yes
o No

Mechanical workshops*
o Yes
o No

Landfills*
o Yes
o No

Cultivated Fields*
o Yes
o No

Chemical warehouses*
o Yes
o No

SECTION 7 - Possible EDC exposure due to parents' occupation

Have you ever worked in one of these sectors or industries?
o agriculture sector
o rubber industries
o plastic industries
o petrochemical plants
o metallurgical plants
o electronics industries
o beauty sector
o dyes industries
o incinerators

SECTION 8 – Possible EDC exposure due to lifestyle

Do you reuse plastic bottles multiple times to store drinks or food?+
o Yes
o No

Do you reuse disposable plastic containers?+
o Yes
o No

Do you heat food in plastic containers in the microwave?+
o Yes
o No

Do you store warm food in plastic containers?+
o Yes
o No

Do you use clingfilm?+
o Yes
o No

Do you consume packaged meat/vegetables?+
o Yes
o No

Do you use antiparasitics for animals?+
o Yes
o No

Does anyone in the family smoke at home?+
o Yes
o No

Do you use mosquito spray repellents at home?+
o Yes
o No

Do you use pesticides at home (e.g. insecticides)?+
o Yes
o No

Do you use care sprays at home?+
o Yes
o No

Do you use care creams at home?+
o Yes
o No

Do you use candles/incenses/air fresheners at home?+
o Yes
o No

SECTION 9 - Eating habits

At home, how often do you consume:

Bovine meat
o Never
o Annually
o Monthly
o Weekly

Pork meat

o Never
o Annually
o Monthly
o Weekly

Poultry meat
o Never
o Annually
o Monthly
o Weekly

Sheep meat
o Never
o Annually
o Monthly
o Weekly

Other meat
o Never
o Annually
o Monthly
o Weekly

Processed meat
o Never
o Annually
o Monthly
o Weekly

Smoked food
o Never
o Annually
o Monthly
o Weekly

Fried food
o Never
o Annually
o Monthly
o Weekly

Steamed food
o Never
o Annually
o Monthly
o Weekly

Food cooked in oven
o Never
o Annually
o Monthly

o Weekly

Food cooked in microwave
o Never
o Annually
o Monthly
o Weekly

Grilled food
o Never
o Annually
o Monthly
o Weekly

Canned foods
o Never
o Annually
o Monthly
o Weekly

Soya- derived food
o Never
o Annually
o Monthly
o Weekly

Water in plastic bottles
o Never
o Annually
o Monthly
o Weekly

Water in glass bottles
o Never
o Annually
o Monthly
o Weekly

Tap water
o Never
o Annually
o Monthly
o Weekly

Water from public water sources
o Never
o Annually
o Monthly
o Weekly

SUPPLEMENTARY MATERIALS 2

Oestrogenic Activity in Girls with Signs of Precocious Puberty as Exposure Biomarker to Endocrine Disrupting Chemicals: A Pilot study

Marta **Gea**[1]*, Anna **Toso**[1,2], Giuseppe Nicolò **Bentivegna**[1], Raffaele **Buganza**[3], Enrica **Abrigo**[3], Luisa **De Sanctis**[1,3], Tiziana **Schilirò**[1]

[1]Department of Public Health and Pediatrics, University of Turin, 10126 Turin, Italy
[2]Institut de Recherche en Cancérologie de Montpellier (IRCM), Inserm U1194, Institut régional du Cancer de Montpellier (ICM), Université Montpellier, 34090 Montpellier, France
[3]Unit of Pediatric Endocrinology, Department of Public Health and Pediatrics, Regina Margherita Children Hospital, University of Turin, 10126 Turin, Italy

*Corresponding author:

Marta Gea

marta.gea@unito.it

Department of Public Health and Pediatrics,

University of Turin,

Via Santena 5 bis, 10126 Turin, Italy.

Phone: +39 0116703190

Table S1. Physiological 17β-oestradiol (E2) concentrations in female serum during different lifetime periods and during different phases of the fertile period. Data provided by the Regina Margherita Children hospital.

Period	Phases	E2 (pg/mL)
Pre-pubertal		6.0 – 27.0
Fertile	Follicular phase	12.5 – 166.0
	Ovulatory peak	85.8 – 498.0
	Luteal phase	43.8 – 211.0
Post-menopausal		5.0 – 54.7
